# Neurexin-3 in the paraventricular nucleus of the hypothalamus regulates body weight and glucose homeostasis independently of food intake

**DOI:** 10.1186/s13041-024-01124-3

**Published:** 2024-08-01

**Authors:** Mingdao Mu, Haoyu Sun, Shuyan Geng, Tianxiang Xu, Chuanyao Sun, Zixu Zhang, Sibie Meng, Moyi Li, An Liu, Zhiyuan Yang, Wei Xie

**Affiliations:** 1 School of Medicine, Southeast University, 87 Dingjiaqiao Road, Nanjing, P. R. China; 2 The Key Laboratory of Developmental Genes and Human Disease, Ministry of Education, The School of Life Science and Technology, Southeast University, 2 Sipailou Road, Nanjing, P. R. China; 3 School of Artificial Intelligence, Hangzhou Dianzi University, Hangzhou, P. R. China

**Keywords:** Neurexin-3, PVN, CaMKIIα-expressing neuron, Adipogenesis, Obesity

## Abstract

Neurexin-3 (Nrxn3) has been genetically associated with obesity, but the underlying neural mechanisms remain poorly understood. This study aimed to investigate the role of Nrxn3 in the paraventricular nucleus of the hypothalamus (PVN) in regulating energy balance and glucose homeostasis. We found that Nrxn3 expression in the PVN was upregulated in response to metabolic stressors, including cold exposure and fasting. Using Cre-loxP technology, we selectively ablated Nrxn3 in CaMKIIα-expressing neurons of the PVN in male mice. This genetic manipulation resulted in marked weight gain attributable to increased adiposity and impaired glucose tolerance, without affecting food intake. Our findings identify PVN CaMKIIα-expressing neurons as a critical locus where Nrxn3 modulates energy balance by regulating adipogenesis and glucose metabolism, independently of appetite. These results reveal a novel neural mechanism potentially linking Nrxn3 dysfunction to obesity pathogenesis, suggesting that targeting PVN Nrxn3-dependent neural pathways may inform new therapeutic approaches for obesity prevention and treatment.

**Supplementary Information:**

The online version contains supplementary material available at 10.1186/s13041-024-01124-3.

## Main text

The worldwide prevalence of obesity has escalated dramatically over recent decades, posing a major public health challenge. Genome-wide association studies have identified neurexin-3 (Nrxn3) as a novel obesity risk gene, with polymorphisms significantly associated with increased body mass index and waist circumference across diverse populations [1–4]. Neurexins are presynaptic cell-adhesion molecules that organize trans-synaptic signaling [5, 6]. However, there have been no direct reports on the role of neurexin-3 in regulating energy metabolism or obesity, and the neural circuits and mechanisms through which neurexin-3 influence energy balance remain largely undefined. The paraventricular nucleus of the hypothalamus (PVN) is a critical hub for central regulation of metabolism and body weight [7, 8], yet the role of PVN neurexins in energy homeostasis has not been explored. This study aims to bridge this knowledge gap by investigating the function of neurexin-3 in the PVN and its potential involvement in the neural control of energy balance and obesity development.

**Fig. 1:**
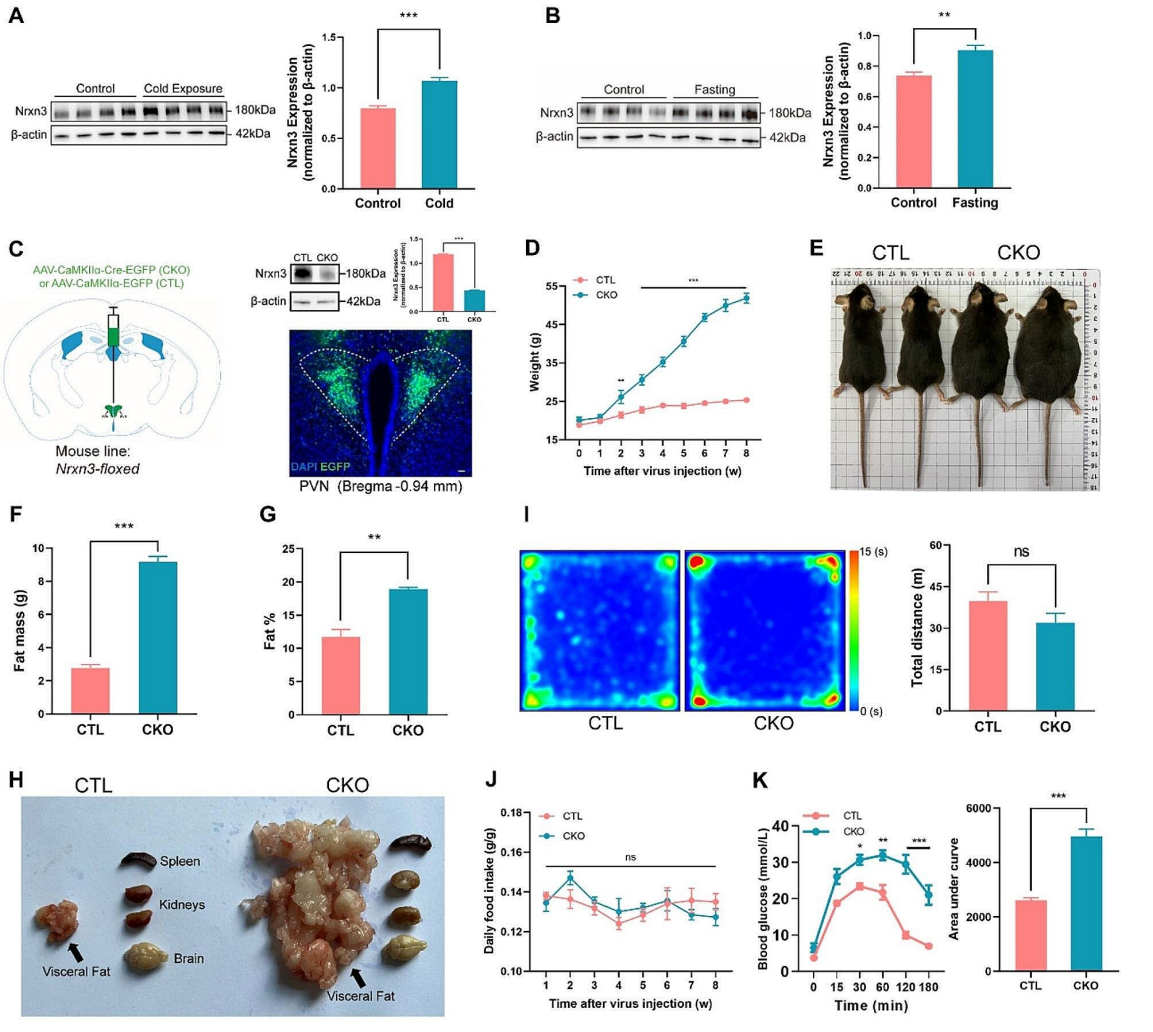
Deletion of Neurexin-3 in PVN CaMKIIα-expressing neurons regulates body weight, adiposity, and glucose homeostasis. **(A-B) **Western blot analysis indicates upregulated Nrxn3 protein levels in the PVN following cold exposure (4 °C, 3 h, A) or after 36 h of fasting **(B)**, compared to control conditions (*n* = 4 mice per group). ** (C)** Left: Schematic diagram of viral injection. Top right: Western blot analysis of Nrxn3 protein levels in PVN. Bottom right: Representative immunofluorescence staining images demonstrating selective transduction of PVN neurons by AAV9-CaMKIIα-Cre-EGFP injection in Nrxn3-floxed mice (*n* = 4 per group). **(D) **Nrxn3 CKO mice show a progressive increase in body weight gain compared to controls (*n* = 5 per group). ** (E)** Representative images of the mice two months post-virus injection. ** (F-G)** Body composition analysis two months after virus injection reveals increased fat mass **(F)** and total fat percentage **(G)** in Nrxn3 CKO mice (*n* = 5 per group). **(H) **Representative images of visceral fat and other tissues in CTL and CKO mice. ** (I)** ANY-MAZE software (Stoelting Co.) records the total distance traveled in a 50 cm (L) x 50 cm (W) x 34.5 cm (H) open field arena, which shows no significant change in Nrxn3 CKO mice during the open field test (*n* = 5–6 per group, total duration: 10 min). **(J)** Daily food intake does not differ significantly between Nrxn3 CKO and CTL mice (*n* = 5 per group). **(K)** Blood glucose levels were measured at 0, 15, 30, 60, 120, and 180 min after glucose injection. Mice were fasted for 12 h before the 0-minute measurement (*n* = 5 per group). Data are presented as mean ± SEM. ns, not significant, *P < 0.05, **P < 0.01, ***P < 0.001, as determined by Student’s t-test

We first examined the expression of Nrxn3 in the PVN under different metabolic states. Western blotting revealed that PVN Nrxn3 levels (Anti-Nrxn3, #48004, Cell Signaling Technology) were significantly upregulated following exposure to either cold (4 °C for 3 h) (Fig. 1A) or fasting (36 h) (Fig. 1B), conditions known to activate PVN neurons and stimulate sympathetic outflow [7, 9]. To directly investigate the role of PVN Nrxn3 in regulating body weight, we generated PVN-specific Nrxn3 knockout mice. This was achieved by injecting an adeno-associated virus expressing Cre recombinase under the control of the CaMKIIα promoter (AAV9-CaMKIIα-Cre-EGFP, PT-0137, BrainVTA Co., Ltd., China) into the PVN of Nrxn3-floxed mice (XM708997, Cas9-Nrxn3-CKO, Nanjing University-Nanjing Biomedical Research Institute) (Supplementary Fig. 1A, B and Supplementary Table). Immunofluorescent staining and Western blot analysis confirmed the virus’s selective transduction of PVN neurons and the efficient knockdown of Nrxn3 protein levels in the PVN (Fig. 1C), a key brain region involved in regulating appetite and metabolism [7, 9–11]. Compared to AAV-CaMKIIα-EGFP-injected male mice (hereafter, CTL group), AAV-CaMKIIα-Cre-EGFP-injected male mice (hereafter, CKO group) exhibited progressive obesity, with a ~ 2-fold increase in body weight gain observed two months after AAV injection (Fig. 1D, E). Body composition analysis revealed a ~ 3-fold increase in fat mass, particularly in visceral fat (Fig. 1F, H). Although there was a trend towards increased weight in the spleen and kidney, these changes were not statistically significant (Supplementary Fig. 1C). Indirect calorimetry revealed no significant change in locomotor activity in Nrxn3 CKO mice (Fig. 1I). Importantly, the observed obesity was not associated with hyperphagia, as daily food intake was similar between Nrxn3 CKO and CTL mice (Fig. 1J). Furthermore, Nrxn3 CKO mice exhibited hyperglycemia and glucose intolerance (Fig. 1K), which are hallmarks of obesity-associated metabolic dysfunction. These findings suggest that the obesity in Nrxn3 CKO mice may be primarily due to increased fat formation, rather than hyperphagia or decreased locomotor activity.

In conclusion, our study identifies Nrxn3 in CaMKIIα-expressing neurons of PVN as a novel regulator of energy balance and glucose homeostasis. Selective ablation of Nrxn3 in the PVN resulted in marked obesity and glucose intolerance, despite normal appetite and locomotor activity. Furthermore, PVN Nrxn3 expression was upregulated by cold exposure and fasting, suggesting that Nrxn3 in these neurons may promote thermogenesis and/or lipid mobilization to maintain energy and glucose balance. These findings provide a neural basis for the genetic association between Nrxn3 and human obesity [1, 2].

The precise cellular mechanisms by which Nrxn3 modulates the activity of PVN CaMKIIα-expressing neurons remain to be elucidated. As a synaptic organizer, Nrxn3 may regulate excitatory and/or inhibitory synaptic inputs to these neurons [5], influencing their responsiveness to metabolic cues. Alternatively, Nrxn3 may control the synaptic output of PVN CaMKIIα-expressing neurons to downstream effector regions, such as the sympathetic outflow circuits in the brainstem and spinal cord [7, 9, 12]. Delineating the neural pathways and signaling cascades mediating the metabolic actions of PVN Nrxn3 warrants further investigation using cell type-specific circuit mapping and manipulation tools. Given the sex differences in obesity development and consequences [13], future studies should examine whether PVN Nrxn3 plays a similar role in regulating energy balance in females, since our current study focused on male mice. Furthermore, it will be important to explore the functions of Nrxn3 in other PVN neuronal subtypes and hypothalamic nuclei implicated in body weight control.

In conclusion, our findings reveal that Nrxn3 in PVN CaMKIIα-expressing neurons plays a vital role in controlling body weight, adipogenesis, and glucose metabolism. This offers new understanding of how Nrxn3 dysfunction contributes to obesity, suggesting that targeting Nrxn3-dependent pathways in the PVN might lead to innovative treatments for obesity prevention and management.

### Electronic supplementary material

Below is the link to the electronic supplementary material.

Supplementary Material 1

Supplementary Material 2

## Data Availability

All study data, including analyses, are in this article.

## Abbreviations

PVN: paraventricular nucleus of the hypothalamus
Nrxn3: Neurexin-3
CaMKIIα: Ca2+/calmodulin-dependent protein kinase IIα
CKO: conditional knockout
AAV: adeno-associated virus
