# Supplementary material for: Neurexin-3 in the paraventricular nucleus of the hypothalamus regulates body weight and glucose homeostasis independently of food intake
Source: Mol Brain. 2024 Aug 1;17:49. doi: 10.1186/s13041-024-01124-3 (PMC11295692; doi:10.1186/s13041-024-01124-3)
Supplement: Supplementary file 1 — Supplementary Material 1 [file 13041_2024_1124_MOESM1_ESM.docx]

**Supplementary Figure 1**

**
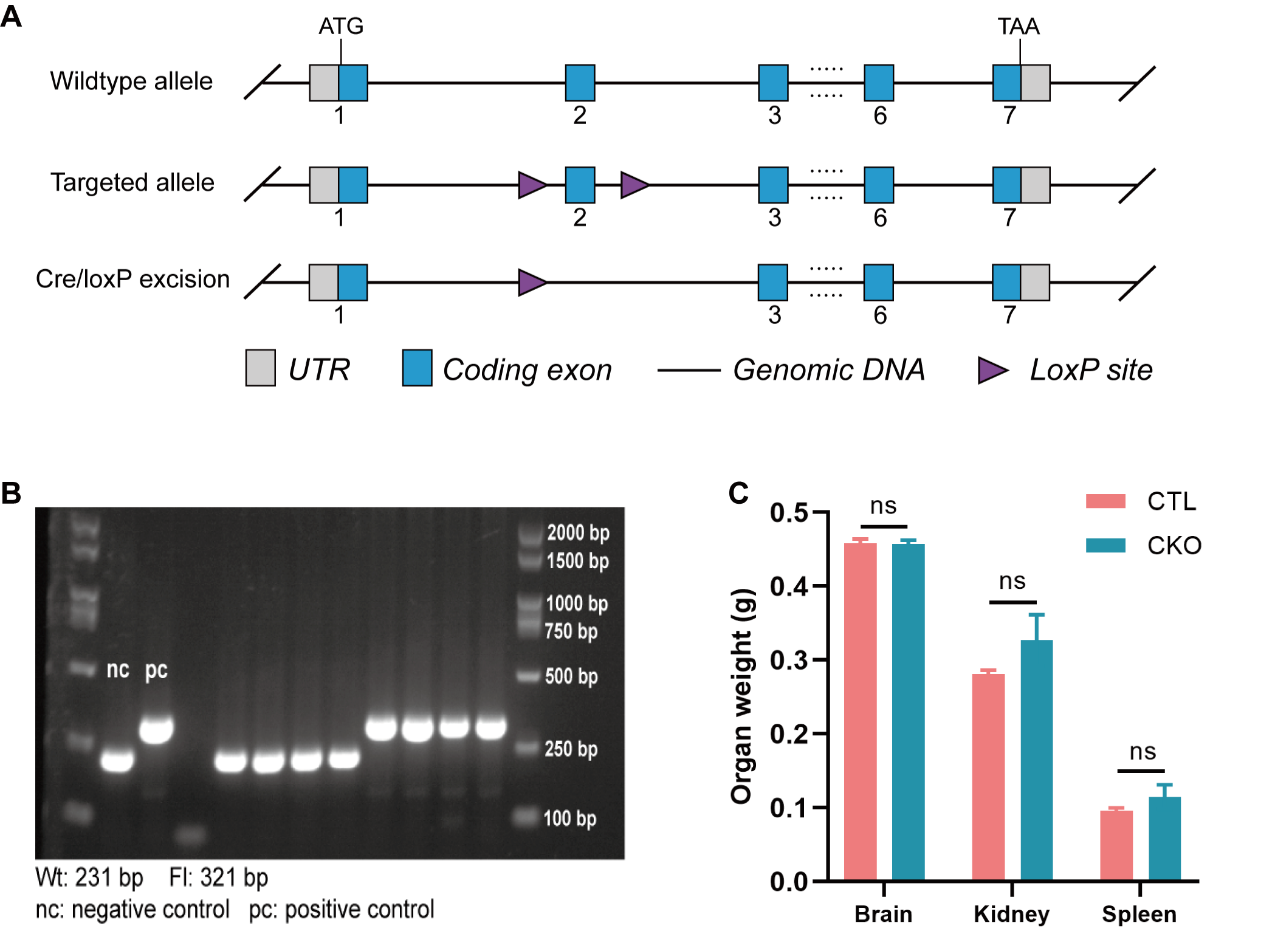
**

1. Strategy for generating Nrxn3-CKO mice; (B) Genotyping results of Nrxn3-CKO mice; (C) Weight of the brain, kidney, and spleen in mice with deletion of Neurexin-3 in PVN CaMKIIα-expressing neurons (CKO) and control mice (CTL). (n=5 per group). Data are presented as mean ± SEM. ns, not significant, as determined by Student's t-test.
