# Supplementary material for: Neurexin-3 in the paraventricular nucleus of the hypothalamus regulates body weight and glucose homeostasis independently of food intake
Source: Mol Brain. 2024 Aug 1;17:49. doi: 10.1186/s13041-024-01124-3 (PMC11295692; doi:10.1186/s13041-024-01124-3)
Supplement: Supplementary file 2 — Supplementary Material 2 [file 13041_2024_1124_MOESM2_ESM.docx]

**Table 1: sgRNA sequences targeting the mouse Nrxn3 gene**

| **sgRNA name** | **sgRNA sequence（5’→3’）** | **PAM** |
| --- | --- | --- |
| 708997-Nrxn3-5S1 | GGGTTGTTGATTACATATGA | AGG |
| 708997-Nrxn3-3S2 | TGATACTCTTATGATCTGCA | AGG |

**Table 2: PCR primer information**

| **Name** | **Sequence** | **Size** |
| --- | --- | --- |
| 708997-Nrxn3-5wt-tF1 | TTCAAAGATGCAGTCCACCAAG | Wt=231bp  Fl=321bp |
| 708997-Nrxn3-5wt-tR1 | ATGAGTGCCACCTCCTAGTGTG |  |
